# Plant Species Classification Based on Hyperspectral Imaging *via* a Lightweight Convolutional Neural Network Model

**DOI:** 10.3389/fpls.2022.855660

**Published:** 2022-04-13

**Authors:** Keng-Hao Liu, Meng-Hsien Yang, Sheng-Ting Huang, Chinsu Lin

**Affiliations:** ^1^Department of Mechanical and Electro-Mechanical Engineering, National Sun Yat-sen University, Kaohsiung, Taiwan; ^2^Department of Forestry and Natural Resources, National Chiayi University, Chiayi, Taiwan

**Keywords:** plant species classification, live-crown features, leaf feature recognition, plant stress detection, dimensionality reduction, convolutional neural network, hyperspectral imaging, deep learning

## Abstract

In recent years, many image-based approaches have been proposed to classify plant species. Most methods utilized red green blue (RGB) imaging materials and designed custom features to classify the plant images using machine learning algorithms. Those works primarily focused on analyzing single-leaf images instead of live-crown images. Without considering the additional features of the leaves’ color and spatial pattern, they failed to handle cases that contained leaves similar in appearance due to the limited spectral information of RGB imaging. To tackle this dilemma, this study proposes a novel framework that combines hyperspectral imaging (HSI) and deep learning techniques for plant image classification. We built a plant image dataset containing 1,500 images of 30 different plant species taken by a 470–900 nm hyperspectral camera and designed a lightweight conventional neural network (CNN) model (LtCNN) to perform image classification. Several state-of-art CNN classifiers are chosen for comparison. The impact of using different band combinations as the network input is also investigated. Results show that using simulated RGB images achieves a kappa coefficient of nearly 0.90 while using the combination of 3-band RGB and 3-band near-infrared images can improve to 0.95. It is also found that the proposed LtCNN can obtain a satisfactory performance of plant classification (kappa = 0.95) using critical spectral features of the green edge (591 nm), red-edge (682 nm), and near-infrared (762 nm) bands. This study also demonstrates the excellent adaptability of the LtCNN model in recognizing leaf features of plant live-crown images while using a relatively smaller number of training samples than complex CNN models such as AlexNet, GoogLeNet, and VGGNet.

## Introduction

Species composition provides basic individual biological features of a landscape and a forest ecosystem. The ability to identify species of individual plants or trees over an inventory plot as well as a forest stand is essential for the automatic mapping of plant distribution, biological diversity, stand structure, and even for diagnosing the dynamics of a forest stand (Lin et al., 2016; Lin, 2019; Santos et al., 2019). The development of plant mapping techniques has the benefit of identifying signals of climate change based on plant phenology (Lin C. et al., 2018) and advanced tree segmentation (Lin C.Y. et al., 2018; Jaskierniak et al., 2021) *via* remote sensing images. Remote sensing images have recently been used to map species distribution mainly according to spectral information with classification techniques. The gaps in/between tree crowns, which tend to be caused by lower crown density, greenness, and background materials, create a challenge for species classification using high-resolution satellite images (Lin et al., 2015a). Image fusion that integrates very high spatial resolution images with atmospherically corrected high spectral resolution can benefit tree crown delineation and improve the mapping (Lin et al., 2015b; Lin C.Y. et al., 2018). However, pixels of the inter- and intra-canopy gaps in a fused image became more significant and increased impact on species crown reflectance (Lin et al., 2015a). Consequently, plant species recognition with remote sensing images becomes a more complicated task involving not merely pixel-based but also object-based approaches. Recently, advanced sensor technology can acquire very high spatial resolution (VHSR) images from various platforms such as in-situ, drone, airborne, and spaceborne for environmental studies. With regard to plant studies, VHSR images are capable of sensing every subtle difference of reflectance in a scale from sub-centimetric to decimetric size allowing better opportunity to reveal detailed features of materials. This is particularly evident in in-situ hyperspectral imaging systems. Moreover, to address the impact of climate change on a vulnerable vegetation community or ecosystem, the dynamics of the community must be derived from the perspective of plant species composition. Therefore, more effort is needed to investigate the problem of developing suitable remote sensing algorithms for classifying a large number of plant species.

During the last decade, most research on plant classification with red green blue (RGB) images was primarily based on extracting leaf features and performing classification with machine learning (ML; Zhang, 2020) classifiers on single-leaf images. The ML classifiers used include support vector machine, K-nearest neighbor, probability neural network, and so on. The methods of leaf feature extraction include polar Fourier transform (Kadir et al., 2013), Canny edge detection (Salman et al., 2017), Fourier transforms (Hossain and Amin, 2010; Khmag et al., 2017), and wavelet decomposition method (Zhang H. et al., 2012), in which the most frequently used features were the color, shape, contour, and texture of leaves. Additional features such as leaf width factor and leaf edge were also used to develop multiscale-distance feature matrixes to improve classification by Beghin et al. (2010) and Hu et al. (2012). As noted, the issues raised in these image-based plant recognition methods are highly dependent on feature engineering and the lack of leaf composition information. In other words, much more effort should be made to achieve noise removal, leaf feature measurements, and texture divergence calculations. The classification seems very dependent on leaf preprocessing.

With the breakthrough of hardware technology, deep learning (DL; Bengio et al., 2017) became the mainstream data processing method in recent years. Among many DL approaches, the convolutional neural network (CNN) is the most popular and representative one in computer vision and imaging processing communities (Ioffe and Szegedy, 2015; Simonyan and Zisserman, 2015; Szegedy et al., 2015; He et al., 2016; Krizhevsky et al., 2017; Wang et al., 2021; Yang et al., 2021). Different from ML methods, CNN can integrate feature derivation, feature learning, and classifier into a single architecture. Many studies have reported that using CNN approaches can produce significantly higher accuracy than using conventional ML ones, as long as with sufficient training data. The reason is that CNN can automatically learn objective, multi-scale, and most discriminative features from raw data without human subjectivity. Following this trend, a few CNN-based plant recognition methods were proposed (Lee et al., 2015; Grinblat et al., 2016; Carranza-Rojas et al., 2017; Lee et al., 2017; Chen et al., 2018; Zhu et al., 2019; Chen et al., 2021). A two-dimensional (2D)-CNN model is adopted in each work to learn the discriminative features from the entire RGB plant images. The spatial relationship of leaf arrangement (phyllotaxy) and overlapping patterns can also be discovered. In other words, various spatial features of interest objects revealed in a VHSR image can be processed by suppressing background materials’ signals and therefore recognized based on the spatial pattern in spectra. This allows us to identify plant species in a way very similar to phytologists with plant morphological features such as leaf color and size, contour, surface, venation, and even phyllotaxy.

Although the current DL approaches demonstrate a certain level of reliability, they still may fail to handle the cases that contain plant species that are similar in appearance, even with enough training data, due to the limited spectral information provided by RGB imaging. If two or more plants have similar outer appearance characteristics, the CNN-based methods may misclassify them. Under such circumstances, it is necessary to use the imaging system providing more delicate spectral information to improve the recognition performance. With the advancement of remote sensing imaging technology, hyperspectral imaging (HSI; Chang, 2013) was developed and widely applied to many topics such as agriculture (Nicolaï et al., 2006; Baiano et al., 2012; Teena et al., 2014; Jung et al., 2015; Marshall et al., 2015; Rapaport et al., 2015; Adão et al., 2017; Gao et al., 2018; Mirzaei et al., 2019; Sun et al., 2019; Sinha et al., 2020; Feng et al., 2021), military defense (Briottet et al., 2006), environment (Zhang B. et al., 2012; Schmitter et al., 2017; Harrison et al., 2018; Abbas et al., 2021), plant phenotyping (Ubbens and Stavness, 2017; Nasiri et al., 2021), and medical imaging (Liu et al., 2007; Fei, 2020). The familiar HSI image contains hundreds of spectral bands ranging from the visible spectrum to the near-infrared (NIR) spectrum so that it can capture the complete spectral characteristics of target objects. Due to its superior spectral resolution, many substances indistinguishable to the naked eye can be recognized. In recent years, hyperspectral cameras have been gradually commercialized. The use of micro hyperspectral cameras for research has become more and more popular. Therefore, using HSI technology to classify plant species has great potential.

Since the use of both HSI and DL techniques for plant species recognition has not been fully explored, in this paper, we conducted a study that adopts hyperspectral plant images as the sample materials and designed a lightweight CNN model to achieve accurate image classification. Firstly, we collect the hyperspectral images from 30 plants of different species with a hyperspectral camera and build a dedicated HSI plant dataset containing 1,500 images. Since the existing CNN-based classification models were designed for the datasets composed of tens of thousands of RGB images of specific objects with a large number of categories, they may not be suitable for the training of our HSI plant dataset. Therefore, this study proposed an improved lightweight convolutional neural network based on the architecture of GoogLeNet (Szegedy et al., 2015) to disclose the issues of species classification through hyperspectral images by deep learning technique. Hyperspectral images have hundreds of bands that are highly correlated, and spectral information of the bands is excessively redundant for vegetation application such as water content modeling (Lin et al., 2012), hyperspectral signal restoration (Lin, 2017; Lin, 2018), and chlorophyll concentration estimation (Lin et al., 2015c; Lin and Lin, 2019). Appropriate feature selection strategies in deriving critical bands for accurate species classification were also explored. Plant classification will be beneficial in diagnosing the stress of individual trees and, therefore the forest. The objectives of this study are:

(1) Applying hyperspectral imaging technique to build a plant species hypercube dataset consisting of 1500 images of plant species to support developing ML models for the plant species classification,
(2) Investigating the feasibility of applying published deep learning architectures to the species classification based on spectral-textural information of plant live-crown images,
(3) Proposing a lightweight CNN model to catch plant live-crown features in the hyperspectral images to achieve optimistic classification performance, and
(4) Exploring the appropriateness of feature selection for hyperspectral images in species classification and the influence of using a limited number of spectral bands on classification performance.

## Hyperspectral Data Collection

### Plant Preparation

**FIGURE 1:**
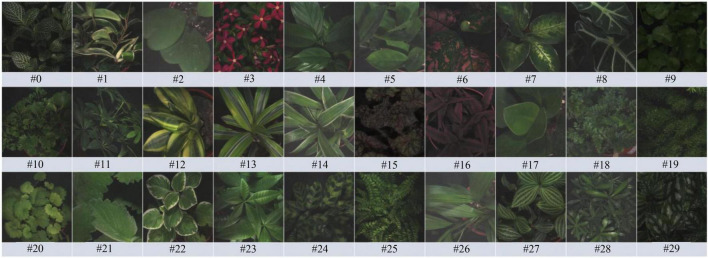
The RGB sample images of the 30 plant species.

As mentioned above, this study aims to recognize plant species based on plant morphology *via* features of leaf color, size, contour, surface, venation, and phyllotaxy. Thirty species of foliage plants from 27 genera and 18 families were collected to produce plant images for analysis. To increase the leaf features and plant geometry diversity in the images, at least 2 or 3 plant individuals were gathered for replications. As shown in Figure 1, leaf features of the plants appeared similar or dissimilar in color, size, venation, and leaf edge. Detailed taxonomy information of the species is shown in Table 1.

**TABLE 1:** The taxonomy information and image samples list of the 30 plant species.

ID	Family	Scientific name	Abbreviation	Number of individuals	Number of full-size images	Number of sub-images
0	Acanthaceae	*Fittonia albivenis*	F.a	2	16	50
1	Apocynaceae	*Hoya carnosa*	H.c	2	16	50
2	Apocynaceae	*Hoya kerrii*	H.k	2	16	50
3	Apocynaceae	*Ammocallis rosea*	A.r	2	16	50
4	Araceae	*Spathiphyllum kochii*	S.k	2	16	50
5	Araceae	*Zamioculcas zamiifolia*	Z.z	2	16	50
6	Araceae	*Aglaonema anyamanee*	A.an	2	16	50
7	Araceae	*Aglaonema commutatum*	A.c	2	16	50
8	Araceae	*Alocasia amazonica*	A.am	2	16	50
9	Araliaceae	*Hydrocotyle verticillata*	H.v	2	16	50
10	Araliaceae	*Polyscias guilfoylei*	P.g	2	16	50
11	Araliaceae	*Schefflera arboricola*	S.a	2	16	50
12	Asparagaceae	*Sansevieria trifasciata*	S.t	3	24	50
13	Asparagaceae	*Dracaena marginata*	D.m	2	16	50
14	Asparagaceae	*Chlorophytum comosum*	C.c	2	16	50
15	Begoniaceae	*Begonia cathayana*	B.c	3	24	50
16	Bromeliaceae	*Cryptanthus bivittatus*	C.b	3	24	50
17	Clusiaceae	*Clusia rosea*	C.r	2	16	50
18	Davalliaceae	*Davallia griffithiana*	D.g	2	16	50
19	Haloragaceae	*Myriophyllum aquaticum*	M.a	2	16	50
20	Lamiaceae	*Glechoma hederacea*	G.h	2	16	50
21	Lamiaceae	*Plectranthus amboinicus*	P.am	2	16	50
22	Lamiaceae	*Plectranthus amboinicus cv.*	P.a.cv	2	16	50
23	Malvaceae	*Pachira aquatica*	P.aq	2	16	50
24	Marantaceae	*Calathea lancifolia*	C.l	3	24	50
25	Nephrolepidaceae	*Nephrolepis exaltata*	N.e	2	16	50
26	Orchidaceae	*Spathoglottis plicata*	S.p	2	16	50
27	Piperaceae	*Peperomia puteolata*	P.p	2	16	50
28	Podocarpaceae	*Podocarpus macrophyllus*	P.m	3	24	50
29	Urticaceae	*Pilea cadierei*	P.c	2	16	50

### Plant Image Acquisition

**FIGURE 2:**
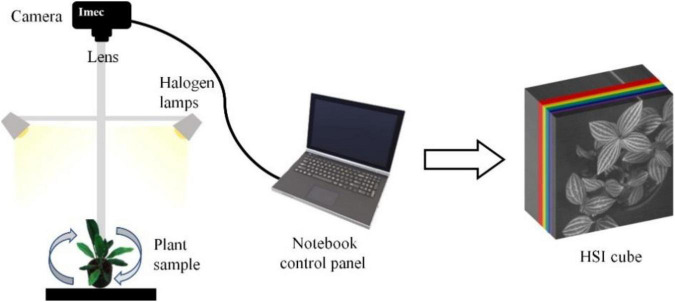
Illustration of plant image acquisition.

The IMEC Snapscan VNIR B150 imaging system^1^ was used to capture the hyperspectral images of the species. This system composes of the spectral image sensor, HSI camera, optics, and some other components that can acquire hypercube datasets up to a full-image size of 3,600 × 2,048 pixels covering a spectrum range from 458 nm to 913 nm. The system’s spectral and radiometric resolutions are 2.8 nm (equivalent to 161 bands) and 10 bits. In the image acquisition, the camera is mounted on a tripod facing downward to the plant at a distance of 40 and 60 cm. Two 50 w/12 V halogen lamps were deployed, one on each side of the plant at a 45-degree elevation angle from the horizontal plane. A black material was used to minimize the background/neighboring material reflectance effects on the target reflectance. The aperture of the camera was set to f5.6 for every single snapshot. Due to the vertical and horizontal variations of the leaves locations, changing the orientation of the plant led to changes in light intensity over the crown area and therefore helped to increase the diversity of the sample images. With the fixed positions of the two light sources, the plant was set to rotate 90 degrees to generate diverse hyperspectral images of the same plant. The image-acquisition scheme is shown in Figure 2. Accordingly, the snapshot acquisition produced a hypercube raw image with a dimension of 1,200 rows × 1,200 columns × 161 bands, and a dynamic range of 10 bits. With the combination of two camera-target distances and four plant orientations, eight HSI raw images of every individual plant of the 30 species were acquired. Due to the significant noise in the wavelengths at both ends of the sensor, the raw image was spectrally subset to 147 bands with a spectrum range of 468–898 nm for the analysis.

#### Data Calibration

To eliminate the impact of inconsistent image quality caused by the environmental factors, such as different illuminations or sensor response, each acquired HSI raw image *Ro* was calibrated with the formula to derive the HSI reflectance image *R_f_*:


(1) $$R_{f}=\frac{R_{o}-I_{B}}{I_{w}-I_{B}}*100\%,$$

where *I_B_* denotes the dark reference image with 0% reflectance recorded with the lens closed, and *I_w_* presents the white reference image with more than 95% reflectance recorded with white a Teflon panel.

#### Hypercube Dataset Preparation

**FIGURE 3:**
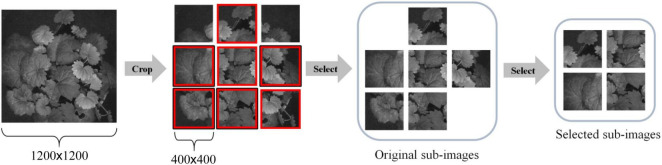
An illustration of sub-image generation.

**FIGURE 4:**
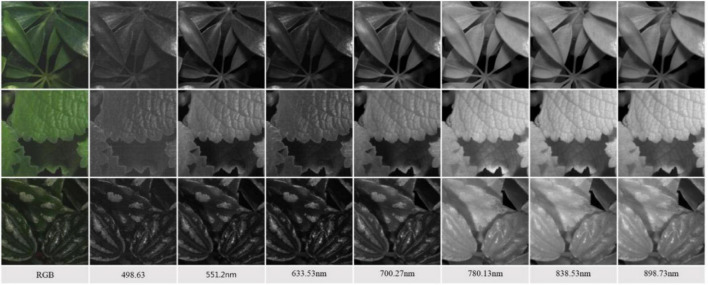
Examples of the reflectance sub-images of plant species (Top: S.a, #11; Middle: P.am, #21; Bottom: P.c, #29) at some selected wavelengths in the visible-NIR region.

To increase the total number of images for DL and reduce the computational complexity of training a CNN model, we adopted the following steps to segment a large image into multiple smaller sub-images. First, each 1,200 × 1,200 HSI reflectance image is evenly segmented into nine non-overlapping 400 × 400 sub-images. Then, those sub-images with a noticeable shadow or insufficient leaves, e.g., the leaf/background ratio does not exceed 60%, were removed. As a result, 50 sub-images were inspected and retained for each species, and a total of 1,500 HSI reflectance images (hereafter hypercube images) were generated for the study. It is worth noting that each sub-image still retains sufficient spatial information of which type of plant it belongs to. The overall process is illustrated in Figure 3. After that, the images were randomly divided into training and test datasets. The former contains 1,200 hypercube images, and the latter has 300 ones. Figure 4 shows some example images of the species at some selected wavelengths in the visible and NIR regions. The reflectance and the features of leaves are retained in each band of the hypercube image.

### Deriving Representative Spectra of the Plant Species

In remote sensing, a reflectance curve is typically representative of the spectral behavior of an object. In addition to the deep learning approach, this study also investigates the spectral reflectance of the plant species. The representative reflectance at each wavelength of the visible-NIR region is determined as the average of the plant leaves. To do this, it is necessary to eliminate background components to extract the region-of-interest (ROI), referred to as leaf regions. The steps to extract the representative reflectance curve of the species are described as follows.

**FIGURE 5:**
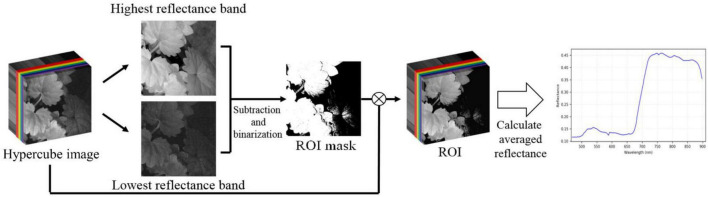
An illustration of leaf region extraction and spectral reflectance generalization of plant species.

First, two particular bands with the largest globally average reflectance, abbreviated maxBand, and the smallest globally average reflectance, abbreviated minBand, among all bands of a hypercube image were identified. Second, a different image of the two specific bands is determined as maxBand – minBand. Third, a thresholding method (Ma et al., 2015) is used to differentiate the different images into two parts, i.e., the region-of-interest vs. the background. The threshold value was set to be 0.3, determined based on the experience. Fourth, a regional-averaging model is adopted to the hypercube image to calculate the average reflectance of every pixel in the ROI. Fifth, the representative reflectance of a particular wavelength of a Plant Species is generalized as the mean of all the corresponding average values of the 50 hypercube images of the species. Finally, the full-wavelength reflectance Spectra of the species are restored by assembling the representative reflectance at every wavelength. Figure 5 illustrates the overall procedures for deriving the generalized reflectance curve.

## Methods

### The Lightweight Convolution Neural Network Architecture

**FIGURE 6:**
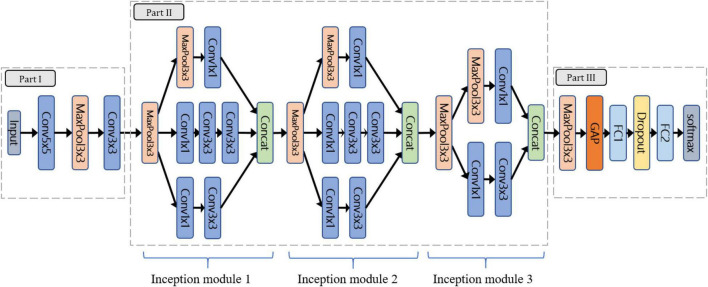
The architecture of the proposed lightweight convolutional neural network (LtCNN). Conv is the convolution layer, Concat means concatenation operation, MaxPool denotes max-pooling layer, GAP stands for global average pooling layer, and FC means fully connected layer. A ReLu function follows every CNN process to increase the network’s nonlinearity.

Considering the limitation of gathering a large number of species and images, this study followed the concept of “compact” in ML to design the lightweight CNN (LtCNN) for better modeling fitting. The LtCNN model is developed by referring to GoogLeNet (Szegedy et al., 2015) and other networks (Ioffe and Szegedy, 2015; Simonyan and Zisserman, 2015; Szegedy et al., 2015). As shown in Figure 6, the architecture of LtCNN is only composed of three parts. The first two are responsible for feature extraction, and the last one is for prediction. The details of those parts are explained below, and the setting of network parameters of the LtCNN model is summarized in Table 2.

**TABLE 2:** Detailed network parameters of the proposed lightweight CNN model.

Parameter	Kernel size	Depth	Strides	Output
Input				200 × 200 × L
Convolution	5 × 5	1	2	100 × 100 × 64
Max pooling	3 × 3	0	2	50 × 50 × 64
Convolution	3 × 3	1	1	50 × 50 × 96
Max pooling	3 × 3	0	2	25 × 25 × 96
Inception module1		2		25 × 25 × 288
Inception module2		2		13 × 13 × 296
Inception module3		2		7 × 7 × 480
Global average pooling	7 × 7	0	1	1 × 1 × 480
FC layer		1		1 × 1 × 196
Dropout (40%)		0		
Output		1		1 × 1 × 30
Softmax		0		1 × 1 × 30
The number of parameters (Input dimension = 6): 1388950				

**Part I:** Part I aims to convert the input image into low-level (or shallow) features as the input of Part II. It comprises two convolutional layers (5 × 5 and 3 × 3) and one pooling layer.

**Part II:** The objective of Part II is to learn the high-level features in a multi-scale manner as the input of Part III. It adopts three “Inception modules” originating from GoogLeNet. Our first two inception modules adopt a 3-path structure and replace the 5 × 5 convolution in the original version with two 3 × 3 ones to reduce the number of parameters while maintaining the same receptive field. The third inception module only adopts a 2-path structure since the size of the feature map has been reduced.

**Part III:** Aims to perform classification *via* the features received from Part II. It uses global average pooling (GAP) to integrate all the features and then applies two fully-connected (FC) layers, one dropout layer, and a Softmax classifier to predict the species of the input image.

### Loss Function

The cross-entropy is selected as the loss function to measure the difference between two probability distributions of the target ground truth and the model’s prediction. It is defined by


(2) $$loss_{CE}=\sum_{c=1}^{C}\sum_{i=1}^{S}-y_{c,i}\log_{2}\left(p_{c,i}\right),$$

where *C* stands for the number of classes, *S* denotes the batch size, *y*_*c,i*_ is a binary indicator, and *p*_*c,i*_ is the predicted probability. In our experiment, we set *S* = 12 and *C* = 30.

### Experimental Setting

The experiments were implemented on the hardware environment with an Intel i7-7700k CPU, 32 GB RAM, and NVIDIA GTX-1080Ti GPU. Three well-known CNN models such as AlexNet (Krizhevsky et al., 2017), VGGNet (Simonyan and Zisserman, 2015), and GoogLeNet (Szegedy et al., 2015) were applied as a referring method for Performance comparison of the Species recognition/Classification. Since AlexNet was designed for the classification of a large number of categories with very deep neural networks, the number of neurons of FC layers was reduced. Specifically, the number of output classes was set to 30 in this study. This model is, therefore, named AlexNetr. Similarly, it is difficult to reach convergence when training the original VGGNet (16 layers) on our plant dataset. The original architecture of VGGNet (16 layers) is therefore simplified by preserving the first eight convolution layers and three FC layers and reducing the number of neurons in the FC layers. It is named VGGNetr in this study. Similar to the LtCNN, a ReLu activation function is applied to improve the nonlinearity.

All the models are trained from scratch without pre-trained parameters or transfer learning techniques. They are implemented on Tensorflow 1.8.0. The size of the input is set to 200 × 200 × *L* for our lightweight model and 224 × 224 × *L* for other CNN models, where *L* denotes the number of selected bands. If we set *L* = 6, the number of parameters of AlexNetr, GoogLeNet, VGGNetr, and the proposed lightweight model are 10865310, 15901982, 10404938, and 1388950, respectively. For data augmentation, we used random crop, random flip in horizontal or vertical to expand the size of the training dataset. For parameter settings, the batch size was set to 12. The learning rate was set to be exponential decay with an initial rate of 10^–3^ and a decay rate of 0.9 for every 5 epochs. The training epoch was assigned as 200, and the optimizer was ADAM. To evaluate model performance, four quantitative metrics were used: overall accuracy (OA), precision, macro F1-Score, and kappa coefficient.

### Feature Selection Methods

The hyperspectral image bands are mostly correlated, particularly those in a similar spectral region. Using full spectral bands for data analysis may lead to the curse of Dimensionality (Hughes, 1968) and increase the computational burden. To achieve a better calculation efficiency while retaining classification accuracy, data dimensionality reduction (Chang, 2013) is required to select critical bands or discriminative spectral features for the Species classification/recognition with DL techniques. The band selection was made *via* two approaches: manual inspection of the reflectance curves and automatic selection based on the spectral heterogeneity of bands. The methods are summarized in Table 3, and the suggested bands with the corresponding wavelength are listed in Table 4.

**TABLE 3:** A summary of the manual and automatic approaches for band selection.

Approach	Description	Source
RGB	Three bands can be used to simulate the normal-color RGB image. The IMEC hyperspectral sensor recommends the bands.	IMEC Snapscan v1.1.2
NIR	Three near-infrared bands are used to simulate a false-color RGB image and are used as a comparison of the normal-color RGB image. Bands are selected according to the reflectance curve, as shown in Figure 7.	Visually inspection
RGB+NIR	The combination of natural-color and false-color images is mentioned above. It is used to compensate for the spectral information absent in each of the two images.	
PCA	The principal component analysis (PCA) transforms the hyperspectral image to principal components (PCs). Only the first six PCs were selected for they retained over 99% energy of eigenvalues.	Gao et al., 2018; Ma et al., 2015.
UBS	The uniform band selection (UBS) is a typical band selection algorithm based on sampling with equal intervals in the whole spectrum. The full range of wavelengths of the IMEC VNIR sensor is divided into 3, 6, and 9 sub-regions. The datasets with 3, 6, and 9 bands are UBS-3, UBS-6, and UBS-9.	Li et al., 2019.
FNGBS	FNGBS stands for the Fast Neighborhood Grouping Band Selection algorithm that partitions the global wavelengths of an HSI cube into M groups based on a coarse-fine strategy and selects the band with the maximum product of local density and information entropy from each group to obtain a subset with M bands for application. In this study, the selected datasets with 3, 6, and 9 bands are abbreviated as FNGBS-3, FNGBS-6, and FNGBS-9.	Wang et al., 2020.

**TABLE 4:** The selected bands and corresponding wavelengths of the hypercube image for species classification.

Approach	Bands	The selected band no.	Representative wavelengths (nm) of the corresponding bands
RGB	3	2/19/39	471.44/535.06/602.94
NIR	3	89/109/126	750.75/799.99/851.04
RGB+NIR	6	2/19/39/89/109/126	471.44/535.06/602.94/750.75/799.99/851.04
PCA	6	PC1-PC6	A component is a linear transformation of bands as the input
UBS	3	1/74/147	468.63/700.27/898.72
	6	1/30/59/88/117/147	468.63/569.27/664.12/747.22/824.74/898.72
	9	1/19/37/55/74/92/110/128/147	468.63/535.06/594.66/651.37/700.27/761.18/802.71/856.68/898.72
FNGBS	3	36/68/92	591.46/681.80/761.18
	6	14/25/68/92/104/118	514.83/551.20/681.80/761.18/783.39/827.35
	9	15/32/33/69/80/92/103/118/137	520.08/557.84/571.04/685.36/721.58/761.18/780.53/827.35/871.66

## Results

### Use of the Visible-Infrared Spectra Variation of Plant Species for Classification

**FIGURE 7:**
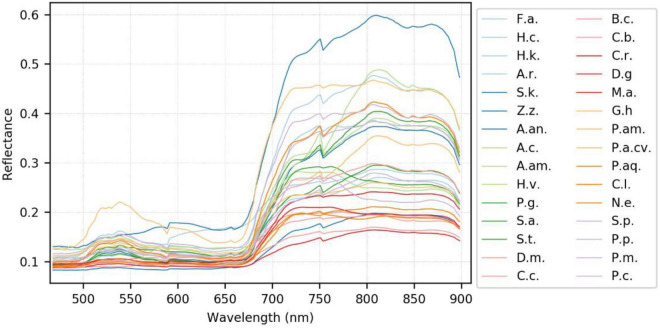
The generalized reflectance curve of the plant species. Refer to Table 1 for the abbreviation of the species.

**FIGURE 8:**
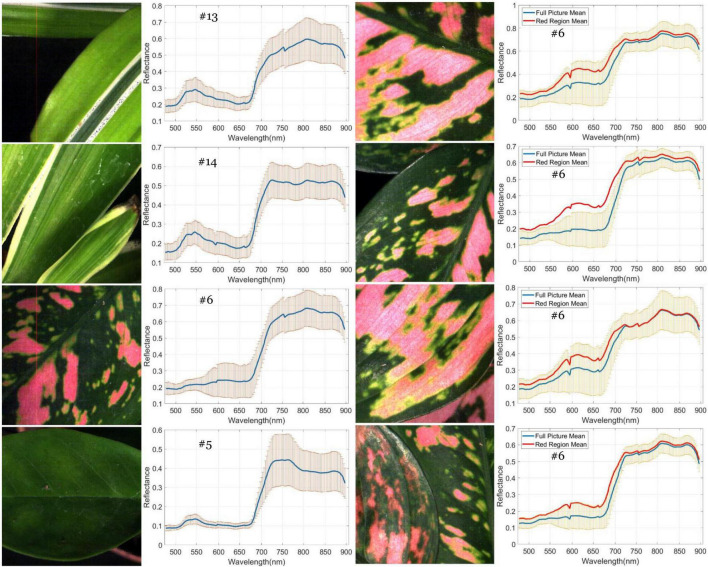
Variation of spectral reflectance in hypercube image of plant leaves. Left: the generalized mean curve and standard deviation ring of the whole leaf pixels for species D.m (#13), C.c (#14), A.an (#6), and Z.z (#5). Right: the difference between the generalized mean curve of the red leaf pixels and that of the global leaf pixels for species A.an. The ring overlapped with the mean curves is the SD of the global leaf pixels.

A generalized reflectance curve of species leaves (average spectra) is essential for differentiating and labeling pixels in a pixel-based classification. Figure 7 shows the averaged spectra of the ROI regions of the 30 plant species, where the x-axis denotes wavelength and the y-axis indicates reflectance values. Each curve was drawn by averaging the spectral reflectance vectors of all the leaf pixels of that particular hypercube image of a species. As can be seen, the reflectance spectra of all species vary at each of the wavelengths. At the same time, the particular features of green peaks, blue and red valleys, and near-infrared plateau remain evident and visually differentiated. Due to the complicated light environment in leaf pixels and even a natural variety of leaf colors for the same species, the reflectance of the species changed dramatically and consequently showed a wide SD band along the visible-infrared regions. The high variation of reflectance of the same materials will lead to difficulty of species classification using pixel-based methods. For example, In Figure 8, the leaf of species D.m (#13) has a white line feature distributed from the bottom to the top of the leaf rib, but species C.c (#14) has two white stripes on the leaf edges. In contrast to the all-green-leaf image of species Z.z (#5), the red spots randomly distributed over the leaf mesophylls of species A.an (#6) make it more challenging for pixel-based species classification.

As noted in the subfigures on the right column of Figure 8, four hypercube images of species A.an highlight the difference between the global and local mean reflectance curves. The former is derived from every pixel of the whole image (the blue curve), and the latter is derived from those red leaf pixels (the red curve). In the visible region, the red-curve spectra spread far from the blue curve and locate almost close to the border of the standard deviation band of the global mean spectra. In contrast, the red curve in the infrared region distributes very close to that of the blue curve. These subfigures reveal that infrared reflectance of leaves is not correlated to the leaf color but to the chlorophyll contents and water contents in mesophyll tissues. In other words, the infrared reflectance of leaves behaves very similarly for the same species as the standard curve. According to Lin et al. (2012, 2015c), leaf reflectance over the VNIR-SWIR region may affected by leaf water and concentration contents. Their study first highlighted the effect of water stress on chlorophyll concentration estimation and further proposed effective chlorophyll indices to account for the influence of water content to achieve accurate estimation of leaf chlorophyll concentration. Therefore, the dissimilarity in the infrared reflectance among hypercube images indicates the possibility of species difference or physiological stress such as water content shortage. Including near-infrared spectra with visible spectra is beneficial to species classification because leaf pattern features and mesophyll structure are considered simultaneously.

### An Overall Assessment of Species Classification Accuracy for the Four Deep Learning Models

Table 5 shows the accuracy measures of the CNN models performing on 6 different spectral features combinations of the plant hypercube dataset. In the classification with the natural-color RGB bands, the AlexNetr, GoogLeNet, VGGNetr achieved an OA of 76.3, 71.7, and 79.9%, a macro F1-score of 0.758, 0.699, and 0.782, and a kappa value of 0.755, 0.707, and 0.790, respectively. Each of the models had a precision of 0.832, 0.705, and 0.806, which indicates that the AlexNetr model and VGGNetr model showed better adaptability in retrieving information of species leaf features and leaf structure in RGB reflectance and therefore achieved a prediction with lower commission error or false positive than the GoogLeNet model. In contrast, the LtCNN model performed at the best accuracy with a value of OA = 89.7%, macro F1-score = 0.896, and kappa = 0.893 which is correspondingly higher than the previous models by 10–18%, 0.11–0.19, and 0.10–0.19. Using only RGB spectral features, the LtCNN model performed species classification with a commission error around 0.1.

**TABLE 5:** Classification performance of different CNN models applied to different feature selection settings.

CNN model	Feature selection ^¶^	OA (%)	Precision	Macro F1-score	Kappa coefficient
AlexNetr	RGB (3)	76.30	0.832	0.758	0.755
	NIR (3)	60.70	0.637	0.601	0.593
	RGB+NIR (3+3)	82.30	0.836	0.824	0.817
	PCA (6)	70.70	0.716	0.703	0.697
GoogLeNet	RGB (3)	71.70	0.705	0.699	0.707
	NIR (3)	66.00	0.635	0.640	0.648
	RGB+NIR (3+3)	70.30	0.668	0.678	0.693
	PCA (6)	71.00	0.708	0.700	0.700
VGGNetr	RGB (3)	79.70	0.806	0.782	0.790
	NIR (3)	**69.70**	**0.737**	**0.686**	**0.686**
	RGB+NIR (3+3)	84.00	0.88	0.838	0.834
	PCA (6)	78.00	0.803	0.778	0.772
LtCNN	RGB (3)	**89.70**	**0.903**	**0.896**	**0.893**
	NIR (3)	68.00	0.728	0.674	0.669
	RGB+NIR (3+3)	**94.70**	**0.950**	**0.945**	**0.945**
	PCA (6)	**88.00**	**0.898**	**0.881**	**0.876**

*^¶^The numbers in parentheses represent the feature dimensionality used in the classification. The bold number in each column of the four accuracy measures indicates the best performance achieved by the corresponding CNN model with the selected features.*

The accuracy measures decreased significantly when checking with the classification results using three NIR bands or the simulated false-color RGB bands, as mentioned in Table 5. For example, the decrease of OA was 15, 5, 10, and 21% for the AlexNetr model, GoogLeNet model, VGGNetr model, and LtCNN model, respectively. These four DL architectures performed the species classification at an OA between 60 and 70% using only NIR-based false-color images. Obviously, the natural-color RGB images provide more diverse spectral information and inherently spatial information of the species than the false-color NIR images. Such cases are due to some species having a similar leaf mesophyll structure (Hopkins and Hüner, 2004; Lin et al., 2015c) and behaving similarly in the near-infrared bands. As shown in Figure 7, the reflectance in the visible-NIR region varied dramatically and overlapped significantly. This leads to a higher degree of omission and commission error in it. In contrast, the natural-color RGB is supposed to catch leaf color, shape, and surface texture changes and consequently contribute species classification accuracy.

In general, the reflectance of RGB bands is low correlated to the NIR bands. The leaf features derived simultaneously from the RGB natural-color bands (471.44, 535.06, and 602.94 nm) and the NIR false-color bands (750.75, 799.99, and 851.04 nm) are assumed to be of benefit to species classification. However, as noted in Table 5, the kappa coefficient achieved by the GoogLeNet model was 0.693, which is even slightly smaller than 0.707, the performance baseline achieved in the classification using only the RGB natural-color bands. In contrast, the AlexNetr, VGGNetr, and LtCNN models revealed a lively performance as the OA, F1-score, and kappa significantly increased by nearly 5%, 0.05, and 0.05, respectively. This verifies that additional NIR bands in respect to the RGB basic spectral information are beneficial to species classification.

Although the principal component analysis (PCA) method can transform the spectral information of bands in the hypercube image into several components, the classification using most informational details through the four CNN models did not perform better than the RGB bands’ baseline. For example, the F1-score of PCA and RGB for the AlexNetr, GoogLeNet, VGGNetr, and LtCNN was 0.703/0.758, 0.700/0.699, 0.778/0.782, and 0.881/0.896, respectively. The result implies that the PCA is most likely inappropriate for use in the reduction of the dimensionality of hypercube images in the view of species classification *via* DL. Considering the performance improvement of RGB+NIR classification, the linear transformation of hyperspectral bands most likely destroyed the physical properties of the materials in each band, thereby weakening the spatial relationship between the features or different tissues on the leaf which decreases the classification ability of a CNN.

### A Comprehensive Examination of the Species Confusion in the Models

**FIGURE 9:**
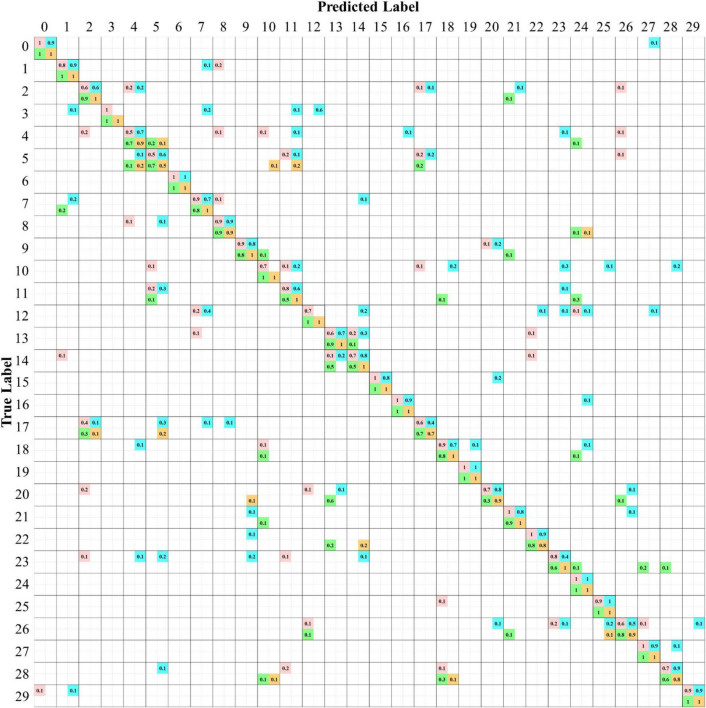
Confusion matrices of species classification of the four CNN models using the six bands of the RGB+NIR dataset. The values in each diagonal entry are the probability of a species image being classified correctly. The numbers in upper/lower off-diagonal entries are the omission rate/commission rate. The numbers highlighted are for the models AlexNetr (pink), GoogLeNet (cyan), VGGNetr (green), and LtCNN (orange).

To illustrate the prediction results of the four CNN models more comprehensively, a confusion matrix is used to examine the confusion among the species for the classification scenario using the RGB+NIR dataset. In Figure 9, the matrix entries contain the numbers of prediction rates of the four models, which are marked with different colored squares. The value of one in diagonal entries specifies the classification of 100% from the view of ground truth. The perfect true-positive rate indicates the superior excellence of a CNN model in describing the leaf features of the plant species. As can be seen, there were 10, 4, 13, and 22 species being classified with 100% of true positive rate for the AlexNetr, GoogLeNet, VGGNetr, and LtCNN models, respectively. As noted in Figure 9, the species *Ammocallis rosea* (A.r, #3) was completely misclassified by the GoogLeNet model. It is mostly recognized as *Sansevieria trifasciata* (S.t, #12) with a false-negative rate of 0.6 while as *Aglaonema commutatum* (A.c, #7), *Schefflera arboricola* (S.a, #11), and *Hoya carnosa* (H.c, #1) with false-negative rate 0.2, 0.1, and 0.1, respectively. Interestingly, this species was recognized accurately by the other three models. Comparing the appearance of species #3, #12, #7, and #1, the flowers of #3 in the hypercube images seem not to work like a feature but a noise in the GoogLeNet model.

**FIGURE 10:**
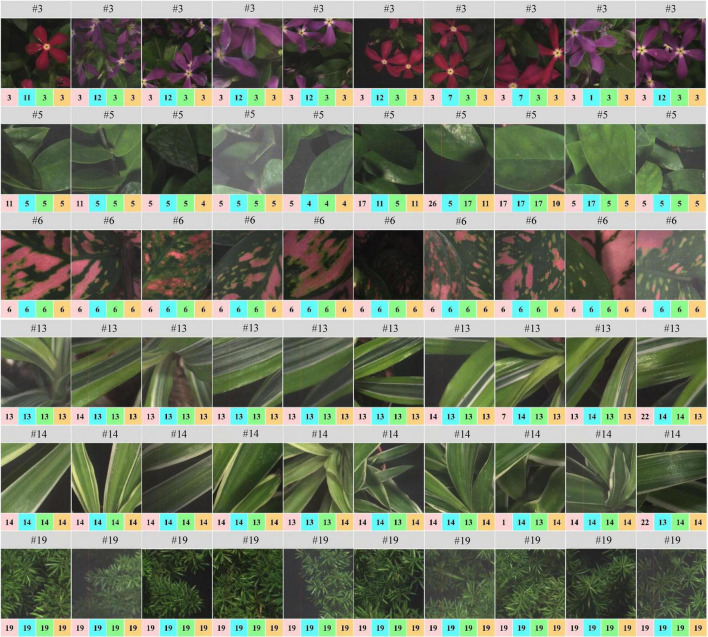
An illustration of species classification by the four CNN models. Species #13 and #14 whose images are entirely recognized by the LtCNN model but eventually misclassified by the other models. The number above each image is the species identity, and the number highlighted by a color box indicates the classified label of the species by the models. Please refer to Figure 8 for the color indication.

Of the 30 plant species, the LtCNN model failed to completely and accurately recognize every image of 8 species, which are *Spathiphyllum kochii* (abbreviated S.a with the species identity #4), *Zamioculcas zamiifolia* (abbreviated Z.z, #5), *Alocasia amazonica* (A.am, #8), *Clusia rosea* (C.r, #17), *Glechoma hederacea* (G.h, #20), *Plectranthus amboinicus cv.* (P.am.cv, #22), *Spathoglottis plicata* (S.p, #26), and *Podocarpus macrophyllus* (P.m, #28) with a true-positive rate of 0.9, 0.5, 0.9, 0.7, 0.9, 0.8, 0.9, and 0.8, respectively. Poorer confidence of classification occurred in species #5 and #17. The false-negative in species #17 is mainly due to the lack of the full leaf shape in the sub-images randomly generated during the convolution, which resulted in a partial leaf and therefore increased the feature similarity of species #17, #5, and #2. Looking into the false-negative classification of species #5, whose images were misclassified as the species #4, #11, and #10 with a rate of 20, 20, and 10%, respectively. These species are visibly differentiated based on leaf margin, surface leathery, and petiole features, but the LtCNN model misclassified 50% of the species. The misclassification is also evident in the other models. This is highly probably due to the inability of retaining leaf margin (serrate and entire), surface leathery, and petiole features during the convolution and pooling processes as the features are too small to detect with respect to the leaf area. Figure 10 illustrates some examples of the confusion in species #3, #5, #13, and #14, and the excellent recognition in species #6 and #19 for the AlexNetr, GoogLeNet, VGGNetr, and LtCNN models. It is also noted that the images of species #13 and #14 were partially misclassified by AlexNetr, GoogLeNet, and VGGNetr models mainly due to the leaf shape similarity; meanwhile, the models missed their heterogeneous features. In contrast, the LtCNN model showed excellence in successfully learning the key features, and therefore the images were classified as the species.

## Discussion

### Band Selection Contributes to Improving the Performance of Species Classification

**FIGURE 11:**
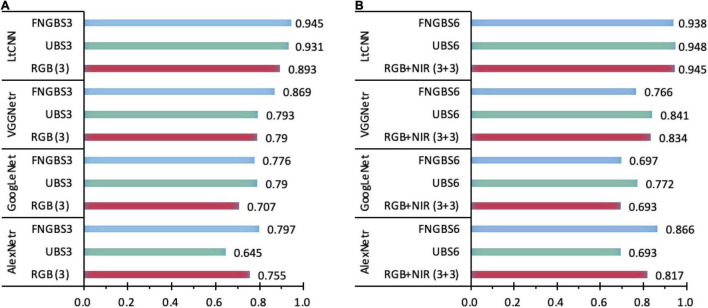
A comparison of the sensitivity of spectral bands for species classification in the four CNN models. Charts **(A,B)** show the kappa coefficients with 3-band and 6-band classifications recommended by the band selection methods. A considerable difference of kappa values in any classifications with different datasets indicates higher sensitivity of the CNN models.

The accuracy figures for the species classification using the dataset with the predetermined bands of RGB, NIR, or RGB+NIR in Table 5 shows the proposed LtCNN is more appropriate than the AlexNetr, GoogLeNet, and VGGNetr for dealing with classification when using a smaller number of species classes. This section examines the contribution of diverse bands in species classification. With regards to 3-band classification, the Fast Neighborhood Grouping Band Selection (FNGBS) method suggested the bands #36, #68, and #92, whose wavelengths are located at the green-edge (591.46 nm), red-edge (681.80 nm), and near-infrared (761.18 nm), while the uniform band selection (UBS) suggested bands #1 (468.83 nm), #74 (700.27 nm), and #147 (898.72 nm) at the regions of blue, red-edge, and near-infrared. As can be seen in Figure 11, the sensitivity of spectral features is evident in each of the four CNN models. With the diverse spectral bands, the kappa changes dramatically. For example, the value dropped by 0.11 for the UBS but raised by 0.042 for the FNGBS in the AlexNetr model. Accordingly, the change rate was equivalent to 15 and 6% of the RGB’s kappa value. In contrast, the GoogLeNet and LtCNN models appeared to be more flexible at catching the spectral features from the three bands suggested by band selection methods. The kappa value was increased nearly by 11∼12% from the baseline of 0.707 for the GoogLeNet model and by 4∼6% concerning the baseline of 0.893 for the LtCNN model for UBS and FNGBS, respectively. Similarly, the VGGNetr model achieved a classification with an increase of kappa value by 10% through the FNGBS suggested bands but failed to improve the performance through the UBS suggested bands.

When the number of spectral bands in a species classification is raised to 6, for example, the bands #14, #25, #68, #92, #104, and #118 selected by FNGBS, three of the models failed to improve the classification performance the exception being the AlexNetr model with an increase of kappa by 0.049 or 6% of the baseline for the RGB+NIR case. Similarly, the classification with a rise in kappa occurred only in the GoogLeNet model when the six bands #1, #30, #59, #88, #117, and #147 recommended by UBS were used for classification. The kappa value was improved from 0.693 to 0.772, and the increase rate was around 11%. The kappa value achieved by the LtCNN model *via* the two band-selection methods is very close to the 3-band case (0.938 vs. 0.945 for FNGBS and 0.948 vs. 0.931 for UBS), this indicates that as long as the band is selected appropriately, using only three bands can achieve a satisfactory classification accuracy.

To summarize, the CNN models appeared to be sensitive to the spectral features of a hypercube image when the number of bands used for species classification is subject to only three spectral bands. For such cases, the FNGBS method works more efficiently and can adapt to AlexNetr, GoogLeNet, VGGNetr, and LtCNN models. And, the LtCNN is the most significant of the four models to achieve reliable and stable classification performance with a minimum number of bands and the most informative spectra. Specifically, the most appropriate spectral features for species classification *via* the LtCNN model are the green-edge (591.46 nm), red-edge (681.80 nm), and near-infrared (761.18 nm).

### Appropriate Dimensionality of Hyperspectral Imaging Images in Recognizing and Classifying Plant Species

**FIGURE 12:**
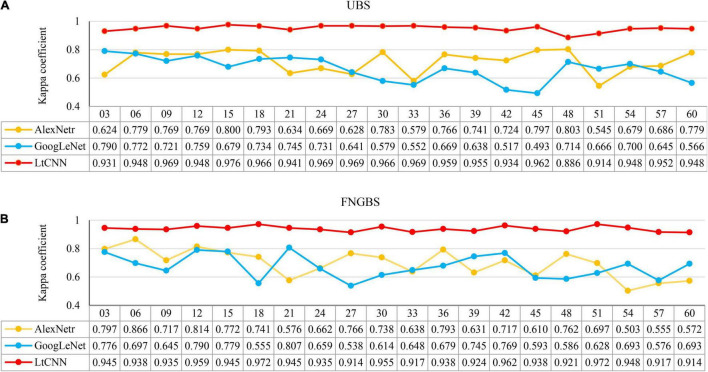
The relationship between the number of bands (M) and produced accuracy when using a subset of hypercube images for classification. Line charts **(A,B)** present the kappa variation for the uniform band selection (UBS) and Fast Neighborhood Grouping Band Selection (FNGBS) method. The numbers below the M list the kappa values of the CNN models.

As noted in Figure 11, the four CNN models revealed diverse sensitivity of spectral bands in species classification. An interesting question arises: Can using more bands help improve accuracy? Or what is the best accuracy achievable by the four models? To address this question this study conducted extended experiments by adding the number of bands progressively up to 60 with an interval of 3 as the input image of species classification for the CNN models. All the models are trained with the parameters mentioned in section “Experimental Setting”, except for the VGGNetr model, because it was unable to handle higher dimensional data under our hardware environment. The adaptability of the AlexNetr, GoogLeNet, and LtCNN models to high-dimensional data is shown in Figure 12.

The x-axis presents the number of bands (M) in each subfigure, and the y-axis shows the corresponding accuracy measure. The yellow, blue, and red curves denote the accuracy trends of the three CNN models, respectively. From the point of view of the species classification, the main observation is that increasing M cannot help to improve accuracy and may even cause worse results. This phenomenon mainly occurred when using AlexNetr and GoogLeNet. The impacts of M on the CNN models are summarized below.

(1). For the cases of UBS, as shown in Figure 12A, the kappa coefficient of AlexNetr starts at 0.624 and increases to 0.779∼0.800 when *M* = 6 to 18. As M increases, the accuracy is no longer improved but becomes unstable. The kappa of GoogLeNet starts at 0.797 and gradually decreases when M increases. For the proposed LtCNN model, the increase of M does not cause a significant change in the classification accuracy. The best value appears at *M* = 15, which is 0.976. After that, the accuracy curve maintains between 0.941 and 0.969. It implies that the amount of spectral information is saturated. On average, the kappa accuracy for the AlexNetr, GoogLeNet, and LtCNN models was 0.717 ± 0.079, 0.666 ± 0.084, and 0.951 ± 0.021, respectively.
(2). Similarly, as shown in Figure 12B for the cases of FNGBS, the trends generated by AlexNetr and GoogLeNet are gradually declining when M increases or fluctuates between 0.503 and 0.866 and 0.538 and 0.807, respectively. On the contrary, the LtCNN model can maintain accuracy between 0.92 and 0.973 and is not sensitive to M. Each of the three models was averaged 0.696 ± 0.096, 0.674 ± 0.081, and 0.940 ± 0.018.
(3). From the point of view of the amount of spectral information, it is evident that using a sufficient number of bands can achieve the highest accuracy. For example, the LtCNN model obtained 0.976 with *M* = 15 selected by UBS in Figure 12A and 0.972 with *M* = 18 recommended by FNGBS in Figure 12B. The other two CNNs models also follow the same fashion. This proves that using hyperspectral imaging for species classification can obtain good results without too many bands.

### Comparison of Conventional Neural Network Models

The AlexNetr and GoogLeNet models produced lower, unstable, and downward accuracy in the plant classification is most likely due to two reasons. Firstly, they were designed and specialized for handling large databases with a large number of categories and training images. Since the tested plant database has only 30 classes and 1,500 images, the training data is relatively insufficient for them. Besides, the nature of the plant image is distinct from the objects’ colors, shapes, and patterns for which the models were originated. This may explain why AlexNetr and GoogLeNet produce lower accuracy performance. Secondly, when M increases, the inter-band correlation of data increases. The input data with excessive redundant information may further interfere with the training process of the more extensive network under insufficient training data. This additionally imposes the difficulty of getting convergence in network training. This may explain why AlexNetr and GoogLeNet produce an unstable performance at different M values. In contrast, the proposed model LtCNN adopts a simplified architecture that is optimized for smaller datasets and significantly performs better than the other two in both accuracy and stability. This emphasizes the importance of designing a dedicated network for processing a particular dataset. And it suggests that as long as the network design is correct, it will not be too sensitive to data redundancy. Meanwhile, it can also be efficient with minimum bands to achieve satisfactory accuracy. Such a conclusion is significant for dealing with hyperspectral images.

### Limitation and Opportunity

The main strength of the proposed plant image classification method is that it uses the abundant spectral information provided by HSI, and uses the “deep features” learned by CNNs for plant species classification. However, this approach suffers from some drawbacks and limitations. Firstly, the cost of hyperspectral cameras is high so it is difficult for it to become widely adopted. Its long-shooting time also limits data acquisition and the possibilities for in-field investigations. Secondly, limited by the existing network architecture of CNN and memory size of GPU, it is hard to use high-resolution HSI images as the material to learn the more comprehensive features. Thirdly, our framework relies on band selection to reduce the data dimensionality. Ideally, we can feed all the bands into CNN and let it learn the discriminative bands automatically. However, limited by the network architecture, memory size, and the amount of training data, it is temporarily impossible to achieve. Finally, if we want to increase the species, except for adding image data to expand the database, it is also necessary to expand the network architecture and retrain the model. This is one of the crucial shortcomings of the current CNN approaches.

Even with these limitations, we are confident that this study will contribute to educational use as well as to the development of plant identification and forest remote sensing. One of the critical findings of this study is that applying only green-edge, red-edge, and near-infrared bands can substantially improve the species classification *via* the proposed model LtCNN. This finding provides an additional opportunity for sensor design specifically for plant applications and therefore benefits the imaging technology development for plant science research and education at a lower investment cost.

## Conclusion

From the point of view of individual tree recognition and mapping, this study applied hyperspectral imaging to build a plant image dataset *via* a VNIR imaging system with a spectral resolution of 2.8 nm and a radiometric resolution of 10 bits. The plant dataset contains 1500 images accounting for the crown and leaf features of 30 species. The plant images show dramatic reflectance values over the spectral range from the visible to the near-infrared region and therefore reveal the dilemma of pixel-based plant classification *via* remote sensing images. Although a pixel-based inspection of plant images reveals that diverse leaf colors increase the difficulty of plant classification using merely visible spectra, the near-infrared reflectance of colorful leaves of the same species remains very similar and behaves homogeneously and stably. In contrast to the variation of visible spectra, the species consistency of near-infrared spectral features provides an optimistic opportunity for plant classification.

According to the results, the complex deep learning architecture of AlexNetr, GoogLeNet, and VGGNetr models are not suitable for plant classification using a limited number of training samples and therefore failed to obtain satisfactory performance when integrating the features in 3-band RGB and 3-band NIR bands. Correspondingly, the best kappa accuracy for these models was 0.817, 0.693, and 0.834. The proposed lightweight conventional neural network, the LtCNN model, however, achieved an optimistic kappa accuracy of 0.945. Interestingly, this novel model has demonstrated its excellence in retrieving critical features from limited training samples through three bands suggested by the fast neighborhood grouping band selection method. The classification using the bands of green-edge (591.46 nm), red-edge (681.80 nm), and near-infrared (761.18 nm), the LtCNN model can achieve a kappa accuracy of 0.945, a value equal to the accuracy of a classification using 6 bands of RGB and NIR. Because the accuracy is very close to the maximum accuracy of 0.976, the best performance with 15 spectral bands of the hyperspectral images, the LtCNN model is concluded to be very efficient and reliable in classifying plant images. It is also concluded that a feature selection should be implemented before applying hyperspectral images to plant classification to reduce training cost and hardware loading significantly.

Many studies developed deep learning techniques for plant classification based on single-leaf images. This study is devoted to exploring an appropriate method for recognizing and classifying plant species according to live-crown and leaf features. Although the hyperspectral imaging technique can provide a hyperspectral dataset with critical spectral features for the application, some false-positive and false-negative errors still occurred in some species by the AlexNetr, GoogLeNet, VGGNetr, and LtCNN models simultaneously. These species are visual recognizably based on the features of leaf margin, surface leathery, and petiole. Developing a new network model with a 3D-CNN module should enhance feature learning in the spectral domain. The ability to retrieve tiny leaf features would also be an essential task for the future.

## Data Availability Statement

The raw data supporting the conclusions of this article will be available at the following link: https://github.com/asufdhlkj456/Plant_Classification_with_HSI_and_DL.

## Author Contributions

CL and K-HL: conceptualization, project administration, methodology, and writing – original draft. K-HL, M-HY, and S-TH: data curation and formal analysis. CL: writing – review and editing. All authors contributed to the article and approved the submitted version.

## Conflict of Interest

The authors declare that the research was conducted in the absence of any commercial or financial relationships that could be construed as a potential conflict of interest.

## Publisher’s Note

All claims expressed in this article are solely those of the authors and do not necessarily represent those of their affiliated organizations, or those of the publisher, the editors and the reviewers. Any product that may be evaluated in this article, or claim that may be made by its manufacturer, is not guaranteed or endorsed by the publisher.

## Footnotes

1
https://www.imec-int.com/en/
